# Supplementary material for: Genomic Patterns of Parallel Divergence Across Demographically Heterogeneous Stickleback Populations in Eastern Canada
Source: Genome Biol Evol. 2026 May 23;18(7):evag083. doi: 10.1093/gbe/evag083 (PMC13372039; doi:10.1093/gbe/evag083)
Supplement: evag083_Supplementary_Data [file evag083_supplementary_data.zip › EC_sup_figures_Final_march9.docx]

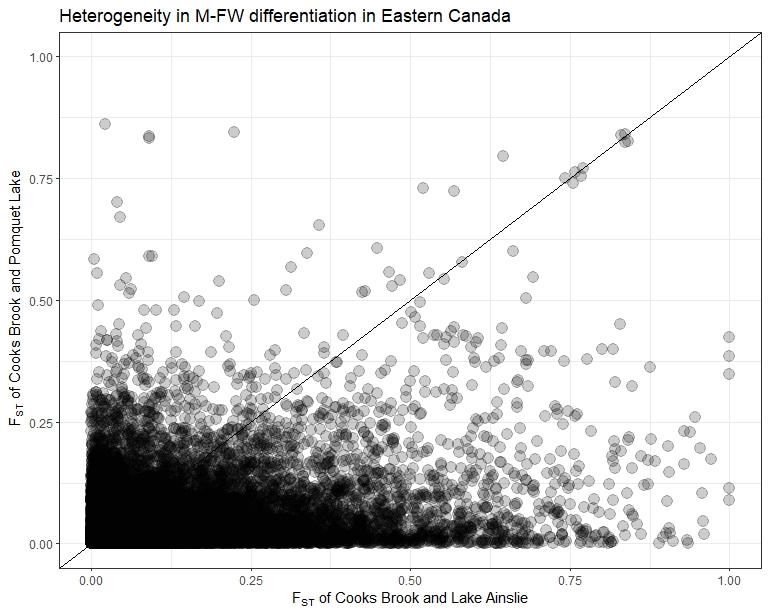

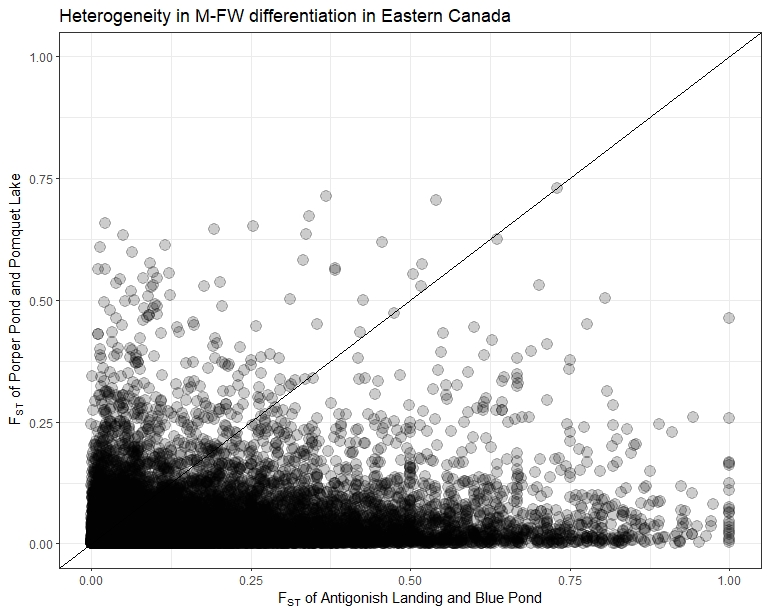


Figure S1. Heterogeneity of M-FW differentiation in Eastern Canada (Newfoundland and Nova Scotia).





Figure S2. Manhattan plots of genome-wide differentiation across marine-marine, marine-freshwater, and freshwater-freshwater population comparisons within, and across, Nova Scotia and Newfoundland.
